# Xp21 contiguous gene deletion syndrome presenting as congenital adrenal hypoplasia: molecular diagnosis and clinical re-evaluation of a pedigree

**DOI:** 10.3389/fendo.2026.1883655

**Published:** 2026-08-28

**Authors:** Donghua Zhang, Wenchun Li, Mei Li, Yinghong Lu, Sisi Ning, Xiafei Liang, Yuling Xie, Yunrong Qin

**Affiliations:** Yulin Women and Children Health Care Hospital, Yulin, Guangxi Zhuang Autonomous Region, China

**Keywords:** CNV-seq, congenital adrenal hyperplasia, congenital adrenal hypoplasia, *NR0B1*, whole-exome sequencing, Xp21 contiguous gene deletion syndrome

## Abstract

**Objective:**

To investigate the molecular etiology in a male child clinically suspected of congenital adrenal hyperplasia (CAH) with negative conventional genetic testing, and to elucidate the genetic characteristics of his pedigree.

**Methods:**

Clinical data from the proband and his family members were collected. Molecular diagnostics proceeded sequentially: initial targeted CAH testing (*CYP21A2* and *POR* sequencing/MLPA, plus targeted *NR0B1* CNV analysis) was followed by whole-exome sequencing coupled with genome-wide CNV analysis.

**Results:**

The proband presented with neonatal cyanosis and hyperpigmentation. Laboratory tests showed markedly elevated ACTH (354.68 pmol/L) and low aldosterone (57.24 pg/mL). Molecular genetic testing identified a hemizygous deletion of approximately 4.44 Mb at Xp21.3-p21.1 (chrX:g.27080000_31520000), encompassing the *NR0B1*, *GK*, *IL1RAPL1*, and *DMD* genes. CNV-seq confirmed that the mother carried a heterozygous deletion of 4.38 Mb in the same region, while the father and four maternal aunts showed normal genotypes.

**Conclusion:**

This study ultimately diagnosed the proband with Xp21 contiguous gene deletion syndrome. The adrenal insufficiency resulted from X-linked congenital adrenal hypoplasia (AHC) due to *NR0B1* haploinsufficiency, rather than CAH. These findings highlight the considerable clinical overlap between AHC and CAH, indicating that CNV analysis of the Xp21 region should be included in the diagnostic workup for male infants with suspected CAH but negative routine genetic testing. This provides a basis for the precise diagnosis and genetic counseling of such patients.

## 1 Introduction

Neonatal adrenal insufficiency with salt-wasting crisis is life-threatening (1, 2). Congenital adrenal hyperplasia (CAH) and clinically similar X-linked adrenal hypoplasia (AHC) are key differential diagnoses. AHC is primarily caused by defects in the *NR0B1* (DAX1) gene located at Xp21.3 (3) and frequently occurs as part of the Xp21 contiguous gene deletion syndrome. In this syndrome, the contiguous deletion of multiple genes can also involve *GK* (causing glycerol kinase deficiency), *IL1RAPL1* (associated with intellectual disability), and *DMD* (causing Duchenne muscular dystrophy), leading to a complex multisystem phenotype (4).

Clinically, reliance solely on hormonal profiles—such as elevated 17-hydroxyprogesterone (17-OHP)—can lead to the misdiagnosis of X-linked AHC as 21-hydroxylase deficiency (21-OHD). Conventional genetic testing for CAH typically targets a fixed set of genes and often fails to detect X-linked *NR0B1* deletions or larger contiguous deletions at Xp21, which may result in a diagnostic deadlock (5). Whole-exome sequencing (WES) combined with copy-number variation sequencing (CNV-seq) enables unbiased, genome-wide detection of both sequence variants and large deletions/duplications, offering a powerful tool for identifying such complex, syndromic cases (6, 7).

The estimated prevalence of AHC is approximately 1 in 70,000 to 600,000 male individuals, although earlier estimates suggested a figure as high as ~1 in 12,500 newborns, which is likely a considerable overestimate (8). In contrast, classic 21-OHD, the most common form of CAH, has an incidence of approximately 1 in 10,000 to 15,000 live births based on newborn screening data, accounting for more than 95% of all CAH cases (9). The clinical presentations of these two conditions overlap considerably, and accurate differentiation relies on molecular genetic testing.

This study presents a male infant initially diagnosed with salt-wasting CAH, who was ultimately identified through clinical and molecular genetic analysis as having Xp21 contiguous gene deletion syndrome, with the adrenal phenotype attributed to AHC resulting from an *NR0B1* deletion. A comprehensive analysis of the familial genetic characteristics and clinical management was conducted.

## 2 Case presentation

### 2.1 Patient information

The proband was a full-term male infant who presented at approximately 12 hours after birth with unexplained vomiting, perioral and facial cyanosis, tachypnea, and generalized hyperpigmentation with decreased skin turgor. Key physical findings included pigmentation over the lips, areolae, and scrotum, along with an enlarged and elongated phallus.

Initial laboratory evaluation revealed significant electrolyte disturbances (hyperkalemia, hyponatremia), markedly elevated cardiac enzymes, and elevated liver enzymes. Hormonal assays showed significantly elevated ACTH and decreased aldosterone, with normal cortisol and 17α-hydroxyprogesterone. Urinary organic acid analysis was notable for increased glycerol. (Complete laboratory and auxiliary examination data are provided in **Supplementary Table 1**).

The mother had epilepsy, managed with levetiracetam and lamotrigine throughout pregnancy. During the second trimester, hospitalization was required for preterm premature rupture of membranes and threatened preterm labor, during which Group B Streptococcus colonization and Chlamydia trachomatis infection were identified. The father was healthy, and the parents were non-consanguineous. All four of the mother’s sisters had no relevant medical history. The mother had a healthy child from a previous marriage.

### 2.2 Methods

Following informed consent, peripheral blood samples anticoagulated with EDTA were collected from the proband, his parents, and four maternal aunts. Genomic DNA was extracted using standard procedures. A tiered molecular diagnostic strategy was employed. First, targeted CAH testing of the proband included capture-based sequencing of CAH-associated genes, in-silico CNV prediction for genes such as *NR0B1* using Kangso CNV software, MLPA for large deletions/duplications in *CYP21A2* and *POR*, and Sanger sequencing of the *CYP21A2* coding region. Subsequently, to comprehensively investigate the etiology, WES (Illumina platform, mean depth ≥100×) was performed on the proband, followed by CNV prediction using ExomeDepth, XHMM, and clinCNV software. Identified variants were filtered against databases such as OMIM and HGMD. Finally, CNV-seq was conducted on family members to determine the origin of the variant.

## 3 Results

### 3.1 Targeted CAH testing results

**Figure 1:**
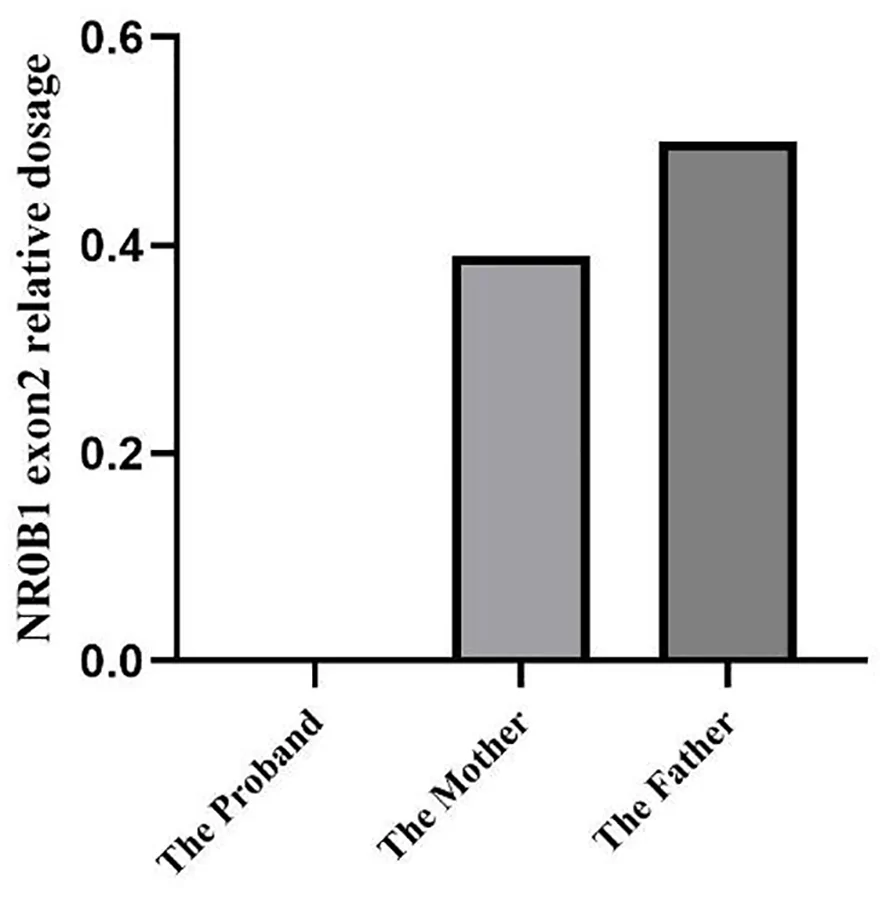
qPCR validation of *NR0B1* exon 2 copy number variations in the proband and family members.

**Figure 2:**
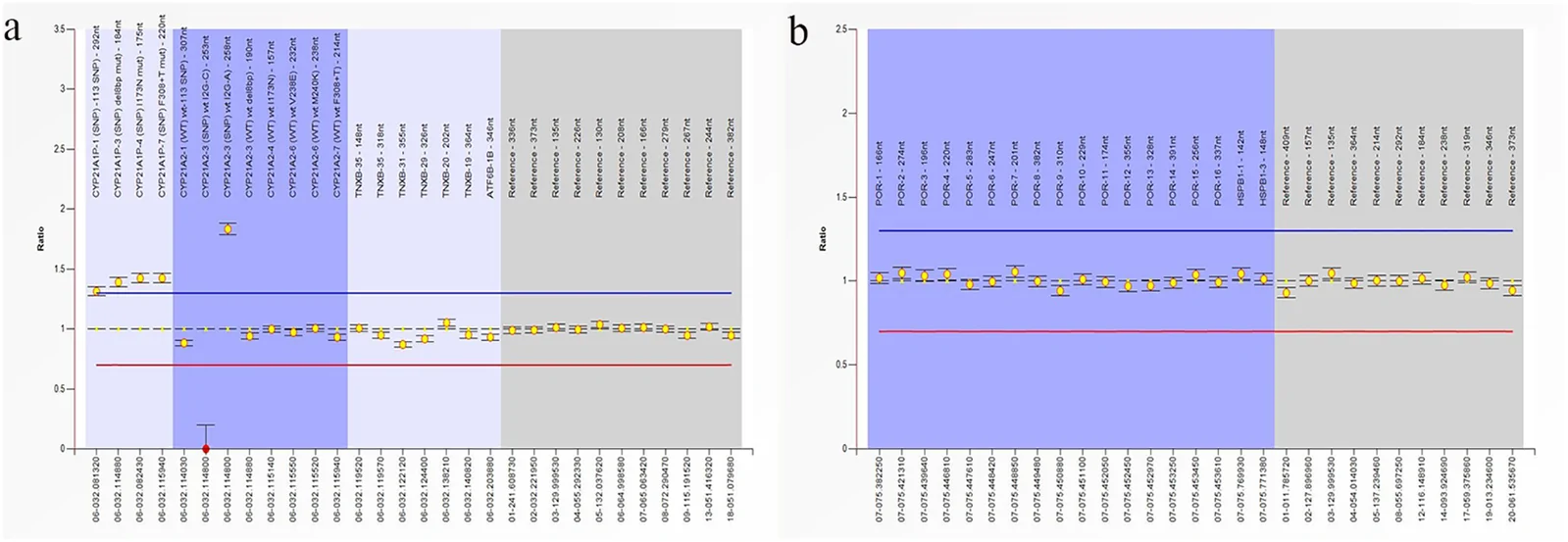
MLPA analysis of the *CYP21A2* and *POR* genes. **(A)** *CYP21A2*; **(B)** *POR*.

No pathogenic small variants (SNVs/InDels) explaining the phenotype were detected. Kangso CNV analysis suggested a potential partial or complete deletion of exon 2 of the *NR0B1* gene (**Figure 1**). MLPA revealed no large deletions or duplications in *CYP21A2* and *POR* (**Figure 2**). Sanger sequencing of *CYP21A2* identified no pathogenic small variants associated with the clinical presentation.

### 3.2 WES and CNV-seq results

**Figure 3:**
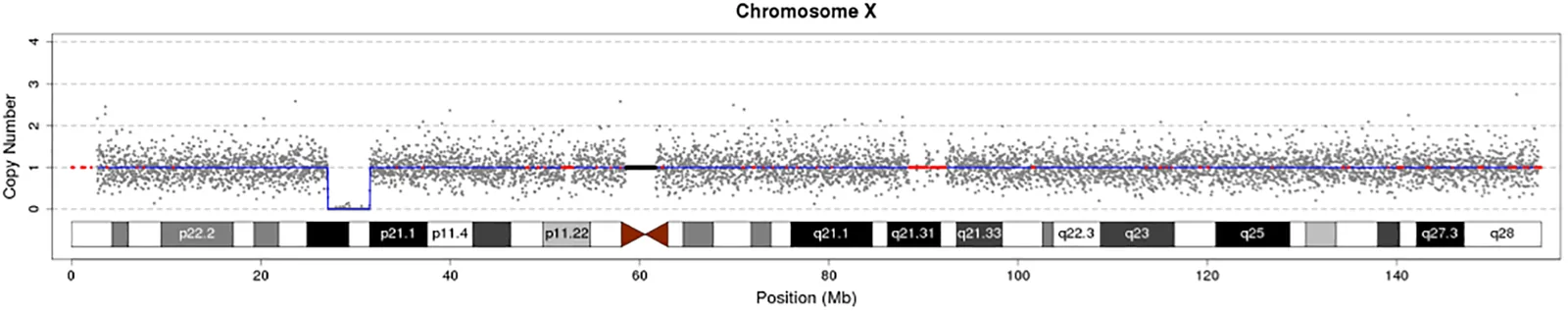
CNV-seq results of the proband.

No pathogenic SNVs/InDels were identified in the proband by WES. CNV prediction based on WES data indicated an approximately 4.018 Mb deletion at chrX:27478905-31497219. This deletion was subsequently validated by CNV-seq analysis, which confirmed a 4.44 Mb deletion in the Xp21.3-p21.1 region of the proband (**Figure 3**), consistent with the initial WES-based prediction.

### 3.3 Familial validation results

**Figure 4:**
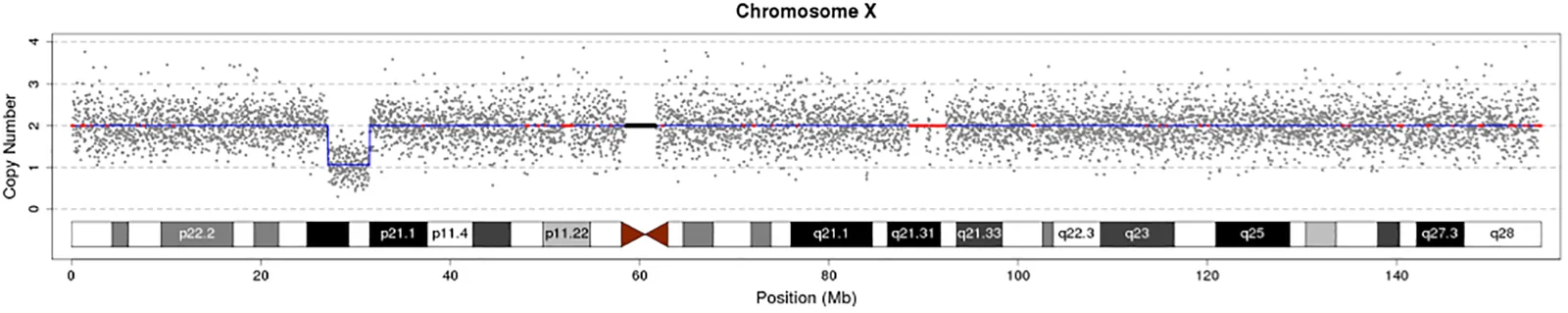
CNV-seq results of the proband’s mother.

**Figure 5:**
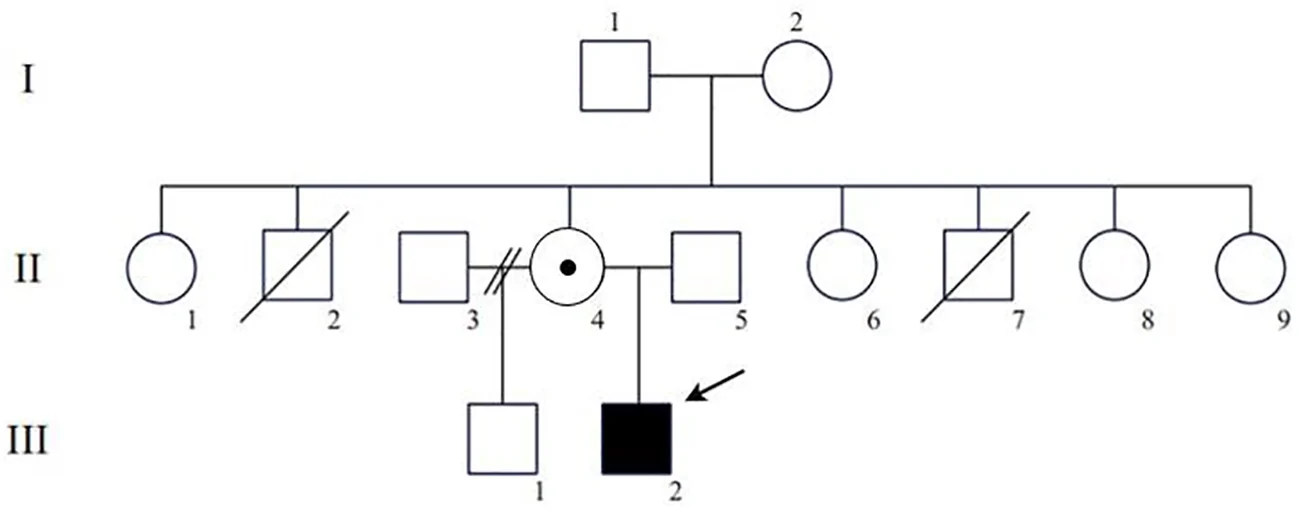
Segregation pattern of the Xp21 deletion in the family. Legend: Squares and circles represent males and females, respectively. Fully filled symbols represent affected individuals. Symbols with a central dot represent unaffected carriers. The arrow indicates the proband. Individuals are numbered in a “generation-individual” format.

CNV analysis of the proband’s mother identified a 4.38 Mb heterozygous deletion within the Xp21.3-p21.2 region (**Figure 4**), confirming her carrier status. In contrast, the father and the four maternal aunts showed normal CNV profiles without the identified deletion. The familial segregation analysis demonstrated that the deletion was inherited from the mother (**Figure 5**), and notably, all four of her sisters were negative for this deletion.

### 3.4 Molecular diagnosis and clinical management

Based on the molecular findings and clinical features, the proband was diagnosed with Xp21 contiguous gene deletion syndrome, presenting primarily as congenital adrenal hypoplasia, with the potential for subsequent subclinical glycerol kinase deficiency, X-linked intellectual disability, and Duchenne muscular dystrophy.

Following diagnosis, the proband received comprehensive management for adrenal crisis, including fluid resuscitation with isotonic saline and 1/2-strength dextrose-saline solutions (with potassium-containing solutions strictly avoided), correction of metabolic acidosis with isotonic sodium bicarbonate as needed, and salt-supplemented formula (sodium chloride 1 g/day). Stress-dose hydrocortisone (100 mg/m²/day intravenously, divided every 8 hours) was initiated immediately. Hydrocortisone was tapered sequentially to 75 mg/m²/day on day 5, 50 mg/m²/day on day 10, and 27 mg/m²/day (6 mg orally every 12 hours) at discharge on day 14. Oral 9α-fludrocortisone (0.05 mg once daily) was added on day 7. By discharge, electrolytes, blood gases, cortisol, and ACTH had normalized. Oral calcium supplementation (300 mg/day elemental calcium) was co-administered during steroid therapy.

A multidisciplinary follow-up protocol was established, encompassing endocrine surveillance for adrenal function, growth, and pubertal development; neurodevelopmental assessment for the *IL1RAPL1* deletion; cardiac and neuromuscular monitoring for the *DMD* deletion; and metabolic evaluation for the *GK* deletion.

## 4 Discussion

AHC is a rare X-linked recessive disorder caused by pathogenic variants in the *NR0B1* gene. This defect leads to impaired adrenal cortex development and steroidogenesis, resulting in glucocorticoid and mineralocorticoid deficiency (10). AHC often presents as the initial manifestation of Xp21 contiguous gene deletion syndrome. Infants typically present acutely within the first weeks to months of life with signs of adrenal crisis, including vomiting, hypoglycemia, and severe electrolyte imbalances (11). However, accumulating evidence indicates considerable variability in clinical manifestations, with some patients presenting at an earlier age or exhibiting atypical features (12). Genotype-phenotype correlations in *NR0B1* mutations remain elusive, and the onset of adrenal insufficiency can vary even among family members carrying the same variant. Beyond adrenal insufficiency, *NR0B1* deficiency is also associated with hypogonadotropic hypogonadism (HH), which typically manifests at puberty with delayed or absent pubertal development (8).

This case illustrates the diagnostic challenge in distinguishing AHC from CAH. Despite a classic salt-wasting presentation, the proband’s normal 17-OHP level was inconsistent with 21-OHD, the most common form of CAH, raising initial doubt. After conventional testing of *CYP21A2* and *POR* genes was negative, the diagnostic strategy shifted to broader genetic analysis. Integrated WES-CNV analysis ultimately identified an Xp21 deletion encompassing *NR0B1*, confirming the diagnosis of X-linked AHC.

The complete deletion of the *NR0B1* (DAX1) gene was the fundamental cause of the life-threatening adrenal failure in the neonatal period, consistent with its central role in adrenal development (13). Deletion of the *IL1RAPL1* gene correlates with the observed neurodevelopmental delay, as its defects are a known cause of X-linked intellectual disability (14). The identified *GK* deletion accounts for the patient’s hyperglycerolemia, the hallmark biochemical feature of glycerol kinase deficiency (15). Furthermore, the partial deletion of the *DMD* gene aligns with the persistently elevated serum creatine kinase (CK) levels (16), indicating subclinical myocyte membrane injury. This necessitates long-term surveillance of muscular and cardiac function due to the risk of delayed-onset myopathy or cardiomyopathy; even atypical partial deletions can result in mild or late-onset muscular phenotypes. Although congenital HH can be assessed during the mini-puberty window by measuring gonadotropin and sex steroid levels, long-term endocrine monitoring of pubertal development remains crucial and should also be incorporated into the patient’s multidisciplinary follow-up plan.

This case has clear implications for genetic counseling and family management, establishing an X-linked recessive mode of inheritance. The mother is a heterozygous carrier, conferring a 50% risk for affected sons and carrier daughters in future pregnancies (17). The absence of the deletion in the four maternal aunts strongly suggests a *de novo* event in the maternal germline, substantially lowering the familial recurrence risk, although the possibility of germline mosaicism should still be mentioned during counseling (18). For future pregnancies, prenatal diagnosis via chorionic villus sampling, amniocentesis, or preimplantation genetic testing is strongly recommended. Their genetic condition also indicates a slightly elevated risk of adrenal insufficiency under stress. The management plan consists of lifelong adrenal hormone replacement and a structured multidisciplinary follow-up, including neurodevelopmental assessment, neuromuscular/cardiac monitoring for *DMD*-related complications, and metabolic guidance for glycerol kinase deficiency.

In summary, this case was ultimately diagnosed as Xp21 contiguous gene deletion syndrome in the proband, with adrenal insufficiency stemming from AHC caused by the deletion of the *NR0B1* gene. It highlights the critical diagnostic challenge posed by the striking clinical similarity between AHC and CAH. Accordingly, we recommend integrating Xp21/*NR0B1*-targeted CNV analysis into the diagnostic evaluation of all male infants with clinically suspected CAH and negative standard genetic testing, a critical step to avoid diagnostic delay and guide precise management. Our findings aid in refining diagnostic pathways, informing genetic counseling, and facilitating multidisciplinary care for this rare syndrome.

## Data Availability

The original contributions presented in the study are included in the article/**Supplementary Material**. Further inquiries can be directed to the corresponding authors.

## References

[1] Krysiak R Claahsen-van der Grinten HL Reisch N Touraine P Falhammar H . Cardiometabolic aspects of congenital adrenal hyperplasia. Endocr Rev. (2025) 46:80–148. doi: 10.1210/endrev/bnae026 39240753 PMC11720181

[2] Mallappa A Merke DP . Management challenges and therapeutic advances in congenital adrenal hyperplasia. Nat Rev Endocrinol. (2022) 18:337–52. doi: 10.1530/ey.19.8.16 35411073 PMC8999997

[3] Gau M Iemura R Orimoto R Adachi E Saito Y Yamano H . Congenital adrenal hypoplasia with neurodevelopmental delay due to contiguous Xp21 deletion: a case series with review of literature. Endocrine J. (2026) 73(3):483–93. doi: 10.1507/endocrj.ej25-0265 41285479 PMC12996724

[4] Singin B Donbaloğlu Z Barsal Çetiner E Bedel A Çetin K Akcan Paksoy B . Xp21 contiguous gene deletion syndrome: diagnosis, treatment, and a review of the literature on a rare genetic disorder. J Clin Res Pediatr Endocrinol. (2026) 18(Suppl 1):83–91. doi: 10.4274/jcrpe.galenos.2025.2024-12-4 40103355 PMC13197103

[5] Sadeghmousavi S Shahkarami S Rayzan E Ahmed S Gharalari FH Rohlfs M . A 3-year-old boy with an Xp21 deletion syndrome: a case report. Endocrine Metab Immune Disord Drug Targets. (2022) 22:881–7. doi: 10.2174/1871530322666220201143656 35105298

[6] Qiao Y Lv N Li T Cheng Y Li Y Dong J . Clinically relevant variants detected in Chinese children with global developmental delay/intellectual disability: an exome-wide sequencing study. Genes Dis. (2025) 12:101389. doi: 10.1016/j.gendis.2024.101389 40212794 PMC11982971

[7] Satam H Joshi K Mangrolia U Waghoo S Zaidi G Rawool S . Next-generation sequencing technology: current trends and advancements. Biology. (2023) 12:997. doi: 10.3390/biology12070997 37508427 PMC10376292

[8] Zheng W Duan Y Xia Y Liang L Gong Z Wang R . Clinical and genetic characteristics of 42 Chinese paediatric patients with X-linked adrenal hypoplasia congenita. Orphanet J Rare Dis. (2023) 18:126. doi: 10.1186/s13023-023-02737-y 37237297 PMC10214642

[9] Botelho Barra C Souza Sena G Pereira Oliveira H de Paula Gonzaga A Ferreira Araújo R Ramos Villela T . Lessons of screening two million newborns for congenital adrenal hyperplasia: 10-year experience of the Minas Gerais Public Health Program. J Pediatria. (2025) 101:341–8. doi: 10.1016/j.jped.2024.10.012 39933694 PMC12039377

[10] Choi HS Kwon A Chae HW Suh J Song KC Lee JS . Identification of a novel point mutation in DAX-1 gene in a patient with adrenal hypoplasia congenita. Ann Pediatr Endocrinol Metab. (2021) 26:126–9. doi: 10.6065/apem.2040088.044 34218634 PMC8255865

[11] Pizza A Picillo E Onore ME Scutifero M Passamano L Nigro V . Xp21 contiguous gene deletion syndrome presenting as Duchenne muscular dystrophy and glycerol kinase deficiency associated with intellectual disability: case report and review literature. Acta Myol: Myopathies Cardiomyopathies: Off J Mediterr Soc Myol. (2023) 42:24–30. doi: 10.36185/2532-1900-246 PMC10115399 37091526

[12] Seki Y Kakimoto H Tamada I Oku S Morita S Tokunaga M . Phenotypic variation among four members in a family with DAX1 deficiency. J Pediatr Endocrinol Metabol: JPEM. (2025) 38:1237–40. doi: 10.1515/jpem-2025-0063 40717045

[13] Achermann JC Vilain EJ . NR0B1-related adrenal hypoplasia congenita. In: Adam MP Bick S Mirzaa GM Pagon RA Wallace SE Amemiya A , editors. Genereviews(®). University of Washington, Seattle, Seattle (WA (1993). Copyright © 1993-2025, University of Washington, Seattle. GeneReviews is a registered trademark of the University of Washington, Seattle. All rights reserved. 20301604

[14] Kokkonen H Siren A Määttä T Kamila Kadlubowska M Acharya A Nouel-Saied LM . Identification of microduplications at Xp21.2 and Xq13.1 in neurodevelopmental disorders. Mol Genet Genom Med. (2021) 9:e1703. doi: 10.1002/mgg3.1703 33982443 PMC8683627

[15] Shah A Xu H Kwon HJ Wondisford FE . *In vivo* glycerol metabolism in patients with glycerol kinase deficiency. JIMD Rep. (2024) 65:392–400. doi: 10.1002/jmd2.12452 39512433 PMC11540572

[16] Bi S Dai L Jiang L Wang L Teng M Liu G . Chronic granulomatous disease associated with Duchenne muscular dystrophy caused by Xp21.1 contiguous gene deletion syndrome: case report and literature review. Front Genet. (2022) 13:970204. doi: 10.3389/fgene.2022.970204 36712874 PMC9880252

[17] Stanković S Stanković T Ignjatović M Jakovljević M Andrejević M . Severe neonatal presentation of Xp21 contiguous gene deletion: adrenal crisis and neuromuscular involvement. Acta Myol: Myopathies Cardiomyopathies: Off J Mediterr Soc Myol. (2025) 44:109–11. doi: 10.36185/2532-1900-1417 PMC12599579 41199733

[18] Bernkopf M Abdullah UB Bush SJ Wood KA Ghaffari S Giannoulatou E . Personalized recurrence risk assessment following the birth of a child with a pathogenic de novo mutation. Nat Commun. (2023) 14:853. doi: 10.1038/s41467-023-36606-w 36792598 PMC9932158

