# Supplementary material for: Xp21 contiguous gene deletion syndrome presenting as congenital adrenal hypoplasia: molecular diagnosis and clinical re-evaluation of a pedigree
Source: Front Endocrinol (Lausanne). 2026 Aug 28;17:1883655. doi: 10.3389/fendo.2026.1883655 (PMC13561775; doi:10.3389/fendo.2026.1883655)
Supplement: Supplementary file 1 [file Table1.docx]

Supplementary Table 1. Detailed laboratory and auxiliary examination data of the proband

| Category | Specific Item | Result | Reference Range / Note |
| --- | --- | --- | --- |
| Laboratory Tests | Sodium | 133.4 mmol/L^*^ | 135-145 mmol/L |
|  | Potassium | 6.59 mmol/L^*^ | 3.5-5.5 mmol/L |
|  | Creatine kinase (CK) | 23137 U/L^*^ | < 300 U/L |
|  | Lactate dehydrogenase (LDH) | 1815 U/L^*^ | 120-300 U/L |
|  | α‑Hydroxybutyrate dehydrogenase | 904 U/L^*^ | 72-182 U/L |
|  | CK‑MB | 534 U/L^*^ | < 25 U/L |
|  | Glucose | 2.4mmol/L^*^ | 2.6-7.0mmol/L |
|  | Alanine aminotransferase (ALT) | 44 U/L^*^ | < 40 U/L |
|  | Aspartate aminotransferase (AST) | 188 U/L^*^ | < 40 U/L |
| Hormonal Assays | ACTH | 354.68 pmol/L^*^ | 1.6-13.9 pmol/L |
|  | Aldosterone | 57.24 pg/mL^*^ | 100-800 pg/mL |
|  | Cortisol | 280.71nmol/L | 118-618 nmol/L |
|  | 17α‑Hydroxyprogesterone | 4.76nmol/L | <5-15nmol/L^a^ |
|  | Testosterone | 55.41 ng/dL | 50-300ng/dL |
|  | Estradiol (E2) | <10pg/mL | <30pg/mL^b^ |
|  | Androstenedione | 0.72nmol/L | 0.7-3.5 nmol/L |
| Urinary Metabolite | Glycerol | 625.9 μmol/mmol creatinine^*^ | Markedly elevated |
| Other Investigations | G6PD activity (cord blood) | 3374 U/L^*^ | Elevated |
|  | Congenital Heart Disease Screening | Positive | _ |

Footnotes: *Values outside the reference range; ^a^ Typical neonatal range; ^b^ Typical neonatal upper limit.
